# Atorvastatin Attenuates Bleomycin-Induced Pulmonary Fibrosis via Suppressing iNOS Expression and the CTGF (CCN2)/ERK Signaling Pathway

**DOI:** 10.3390/ijms141224476

**Published:** 2013-12-16

**Authors:** Bo Zhu, Ai-Qun Ma, Lan Yang, Xiao-Min Dang

**Affiliations:** 1 Department of Pulmonary and Critical Care Medicine, the First Affiliated Hospital, Xi’an Jiaotong University, Xi’an 710061, China; E-Mails: zhubo685689@163.com (B.Z.); ylan8@medmail.com.cn (L.Y.); dxming112@163.com (X.-M.D.); 2 Department of Cardiology, the First Affiliated Hospital, Xi’an Jiaotong University, Xi’an 710061, China

**Keywords:** pulmonary fibrosis, atorvastatin, iNOS, CTGF (CCN2), ERK

## Abstract

Pulmonary fibrosis is a progressive and fatal lung disorder with high mortality rate. To date, despite the fact that extensive research trials are ongoing, pulmonary fibrosis continues to have a poor response to available medical therapy. Statins, 3-hydroxy-3-methylglutaryl-coenzyme A reductase inhibitors, known for its broad pharmacological activities, remains a remedy against multiple diseases. The present study investigated the antifibrotic potential of atorvastatin against bleomycin-induced lung fibrosis and to further explore the possible underlying mechanisms. Our results showed that atorvastatin administration significantly ameliorated the bleomycin mediated histological alterations and blocked collagen deposition with parallel reduction in the hydroxyproline level. Atorvastatin reduced malondialdehyde (MDA) level and lung indices. Atorvastatin also markedly decreased the expression of inducible nitric oxide synthase (iNOS) in lung tissues and, thus, prevented nitric oxide (NO) release in response to bleomycin challenge. Furthermore, atorvastatin exhibited target down-regulation of connective tissue growth factor (CTGF (CCN2)) and phosphorylation extracellular regulated protein kinases (p-ERK) expression. Taken together, atorvastatin significantly ameliorated bleomycin-induced pulmonary fibrosis in rats, via the inhibition of iNOS expression and the CTGF (CCN2)/ERK signaling pathway. The present study provides evidence that atorvastatin may be a potential therapeutic reagent for the treatment of lung fibrosis.

## 1. Introduction

Pulmonary fibrosis is a chronic, progressive lung disorder characterized by the swelling and scarring of the alveoli and interstitial tissues of the lung [1,2]. This damage causes patients’ lungs to stiffen and makes breathing increasing difficult. Pulmonary fibrosis is the most common of interstitial lung diseases, and affects over five million people worldwide with a mean survival time of ~three years [3]. Although research trials are ongoing, there is no evidence that any medication can significantly help with this condition [4–6].

Recent studies indicate that oxidative stress plays a critical role in the pathological development of pulmonary fibrosis, as well as in fibrosis in multiple organ systems [7–9]. Markers of oxidative stress have been identified in the lungs of pulmonary fibrosis patients, and aberrant antioxidant activity exacerbates pulmonary fibrosis in animal models [10,11]. Some reports have noted that the pathogenesis of fibrosis is mediated, at least in part, through the generation of toxic reactive oxygen and nitrogen species [12,13]. Extensive focus has been shifted to reactive nitrogen species (RNS) [14]. An overproduction of nitric oxide (NO), resulting from the expression of inducible nitric oxide synthase (iNOS), has been demonstrated to play an essential role in the induction of pulmonary fibrosis in animal models and humans [15,16]. Thus, agents that depress oxidative stress have potential clinical value, and could have additional protective effects against pulmonary fibrosis.

Statins, which are 3-hydroxy-3-methylglutaryl-coenzyme A (HMG-CoA) reductase inhibitors, are clinically used for the treatment of dyslipidemia and the prevention of cardiovascular disease [17]. Recently, experimental and clinical evidence has indicated that the pleiotropic effects of statins involve inhibiting inflammation and oxidative stress [18,19]. It has also been demonstrated that statins can modulate inflammatory and oxidative stress responses through the regulation of iNOS, tumor-necrosis factor-α, nuclear factor kappa B, and matrix metallopeptidase [20–23]. These findings suggested that statins could be beneficial in the treatment of pulmonary fibrosis. More recently, CCN2, a member of the CCN family of matricellular proteins that was also referred to as connective tissue growth factor (CTGF), has not only been considered as a prognostic marker in evolving fibrotic diseases but also as a potential candidate in new approaches to antifibrotic therapy [24–27]. Although there is some evidence documenting the protective effects of statins on pulmonary fibrosis, the molecular signaling pathways that specifically mediate their effects on oxidative stress in pulmonary fibrosis have not been elucidated.

Therefore, the present study was undertaken to investigate the capacity of atorvastatin to attenuate bleomycin-induced pulmonary fibrosis in a murine model. Furthermore, to shed some light on the mechanism of action, we also assessed the expression levels of iNOS and CTGF (CCN2), as well as the activation of the extracellular signal-regulated protein kinases (ERK) pathways.

## 2. Results

### 2.1. Protective Effects of Atorvastatin against Bleomycin Modulated Body Weight and Lung Indices

A significant reduction in the body weight was observed in the bleomycin-treated animals, compared to saline-treated control animals (*p* < 0.05). Atorvastatin administration increased the body weight in animals treated with bleomycin, but there was no obvious difference in the body weight for the bleomycin-treated group. Simultaneously, the increased lung indices (lung weight/body weight) of the bleomycin-treated animals were prominently reduced when treated with atorvastatin (*p* < 0.05). Moreover, no adverse effects were observed in animals administered atorvastatin alone, similar to the profiles of control animals (Table 1).

### 2.2. Atorvastatin Attenuated Bleomycin Mediated Histological Changes

**Figure 1.**
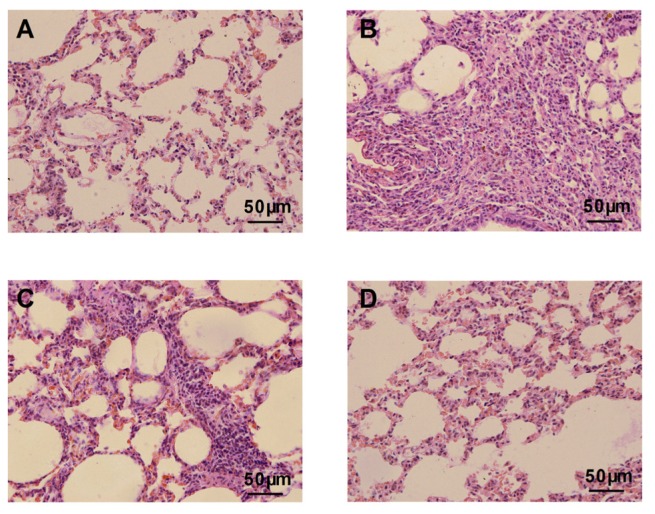
Histological evaluation of atorvastatin on bleomycin-induced lung in rats. (**A**) Lung tissue sections of control animals showing normal lung morphologies: thin lined interalveolar septa with well organized alveolar space; (**B**) Lung tissue sections of bleomycin-induced animals showing distorted lung morphologies: collapsed alveolar spaces with inflammatory exudates, wider and thickened interalveolar septa; (**C**) Lung tissue section of atorvastatin treated animals: lower inflammatory infiltrates with lessened alveolar thickening; and (**D**) Lung tissue section of atorvastatin alone administered animals showing similar morphology with that of control animals. Representative histological section of the lungs was stained by hematoxylin and eosin (×400).

The lung tissue sections of bleomycin-treated animals showed markedly histopathological abnormalities, including disturbed alveolar structure, extensive thickening of the interalveolar septa, and dense interstitial infiltration by lymphocytes, neutrophils, and fibroblasts (Figure 1B). No such variations were observed between the control group and the group treated with atorvastatin alone (Figure 1A,D). In contrast, the atorvastatin treatments provided protection against bleomycin-induced lung tissue distortion. Significant amelioration in the cellular infiltrates and thin-lined alveolar septa were observed in the lung morphologies of the atorvastatin-treated group compared to the bleomycin-treated animals (Figure 1C).

### 2.3. Inhibition Effects of Atorvastatin on Collagen Depositions and Hydroxyproline Content

**Figure 2.**
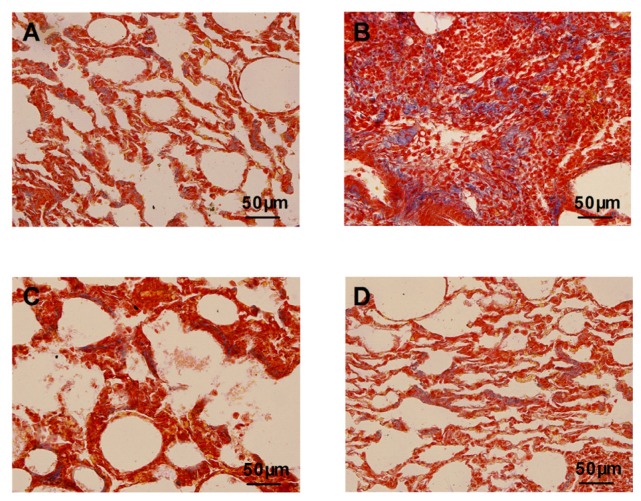
Effects of atorvastatin on histopathogical changes of bleomycin-induced lung with Masson’s trichrome stain (×400). (**A**,**D**) Lung tissue sections of control and atorvastatin alone administrated animals with normal lung morphologies: scarcely deposited collagen in the lung parenchyma; (**B**) Lung tissue sections of bleomycin-induced animals showing dense collagen accumulations: collagen accumulations in lung parenchyma; and (**C**) Lung sections of atorvastatin treated animals showing reduced collagen depositions: reduced alveolar thickening with meager collagen.

**Figure 3.**
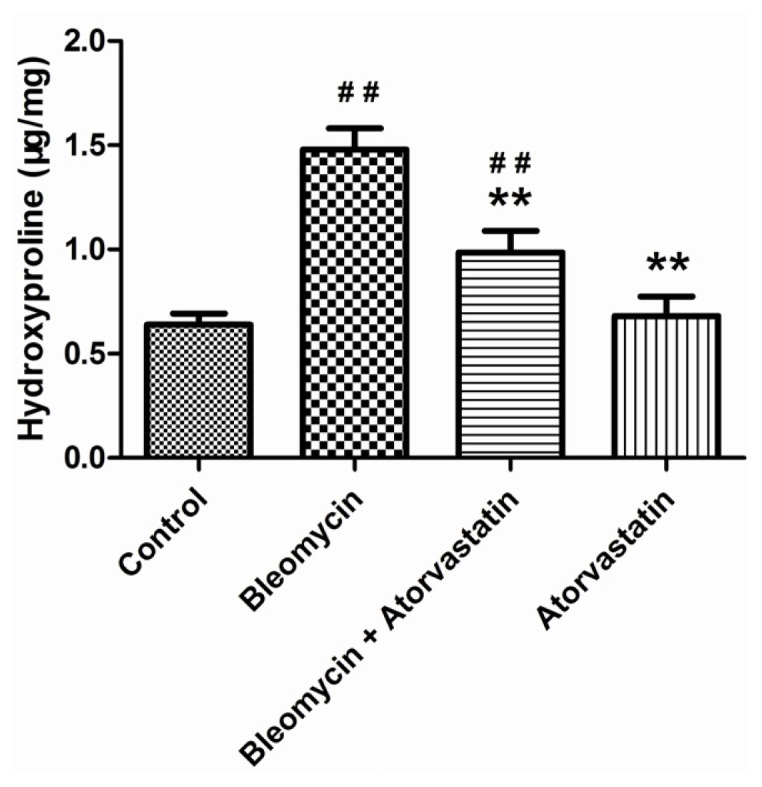
Effects of atorvastatin on the hydroxyproline content in the lungs of bleomycin-induced pulmonary fibrosis rats. Values are given as mean ± SD for groups of eight rats each. ^##^*p* < 0.01 *vs.* the control group; ** *p* < 0.01 *vs.* the bleomycin group.

Masson’s trichrome staining showed that the bleomycin-treated animals had an abnormal collagen deposition and distorted lung morphologies compared with the control animals (Figure 2B). The atorvastatin treatments strongly inhibited the extent and intensity of collagen, compared to the bleomycin treatment (Figure 2C). No such abnormalities were apparent in the control animals and the animals treated with atorvastatin alone (Figure 2A,D). We analyzed the hydroxyproline content of lung tissues, which is considered to be a fibrotic marker of deposited collagen. As summarized in Figure 3, the pulmonary hydroxyproline levels in the bleomycin group were drastically increased, compared to those in the control animals. The atorvastatin treatment reduced the level of hydroxyproline, which correlated with the Masson’s trichrome data. There was no significant difference in the hydroxyproline content of animals administered atorvastatin alone compared to control animals.

### 2.4. Suppressive Effects of Atorvastatin on Oxidative Stress Markers

**Figure 4.**
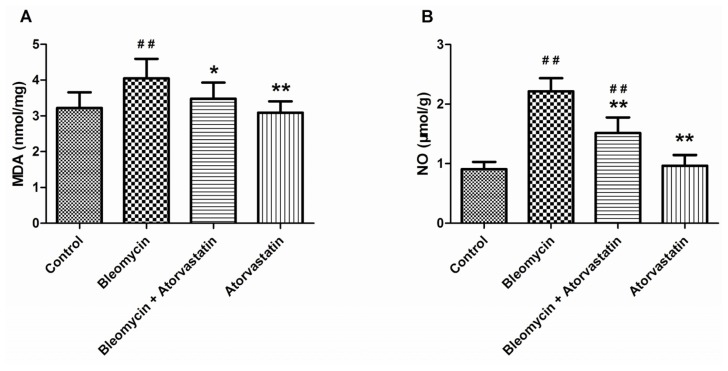
Effects of atorvastatin on the (**A**) malondialdehyde (MDA) and (**B**) nitric oxide (NO) levels in the lungs of bleomycin-induced pulmonary fibrosis rats. Values are given as mean ± SD for groups of 8 rats each. ^##^*p* < 0.01 *vs.* the control group; * *p* < 0.05 and ** *p* < 0.01 *vs.* the bleomycin group, respectively.

To evaluate the oxidative lung injury, the malondialdehyde (MDA) and nitric oxide (NO) concentrations were estimated in the experimental group of animals. As expected, the MDA levels were considerably increased in the lung tissues of bleomycin-exposed animals compared to the control animals. Similarly, the NO levels were significantly enhanced in the bleomycin-treated animals, compared to the control animals. Treatment with atorvastatin reduced the levels of MDA and NO, compared to the bleomycin treatment. There was no significant difference between the control animals and the animals treated with atorvastatin alone (Figure 4).

### 2.5. Down-Regulation of iNOS and CTGF (CCN2) Expression by Atorvastatin Treatment

**Figure 5.**
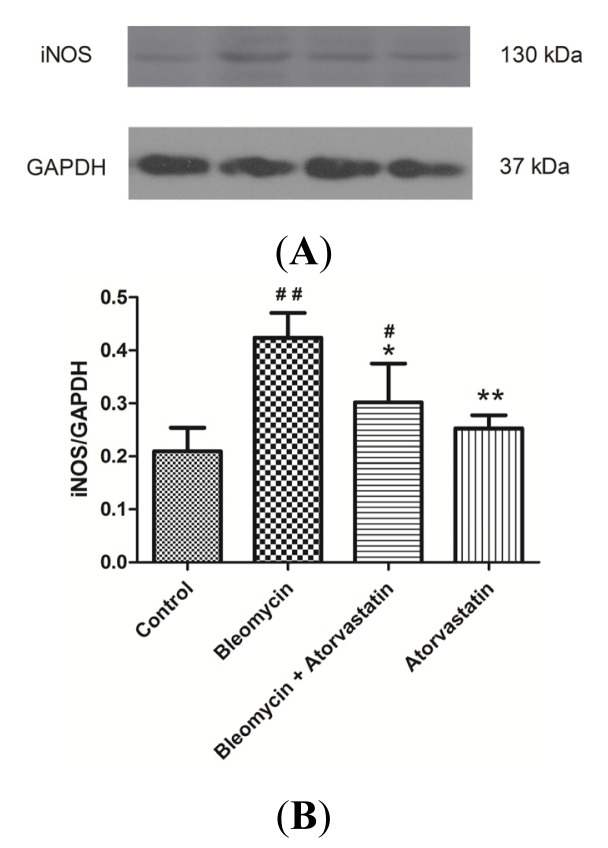
Western blot analysis of iNOS levels in the lungs of bleomycin-induced pulmonary fibrosis rats. The increased levels of iNOS protein expression were significantly inhibited by administration of atorvastatin. (**A**) Representative blots are shown and protein size is expressed in kDa; and (**B**) Densitometric quantification data are expressed as the intensity ratio of target proteins to GAPDH (mean ± SD, *n* = 5). ^#^*p* < 0.05 and ^##^*p* < 0.01 *vs.* the control group, respectively; * *p* < 0.05 and ** *p* < 0.01 *vs.* the bleomycin group, respectively.

Previous studies have shown that bleomycin can induce iNOS over-expression, thereby leading to excessive NO release. To determine whether atorvastatin inhibits NO production via the suppression of iNOS expression, the iNOS protein levels were measured by western blot analysis. Compared to the control group and atorvastatin group, there was a marked increase in iNOS protein levels in the bleomycin group. The atorvastatin treatment significantly attenuated the increase of iNOS protein in the lung tissue of the bleomycin-treated animals (Figure 5A). The results suggested that atorvastatin inhibits NO production via the suppression of iNOS expression in rats with bleomycin-induced pulmonary fibrosis rats. The quantitative analysis of Western blots for iNOS expression is depicted in Figure 5B.

**Figure 6.**
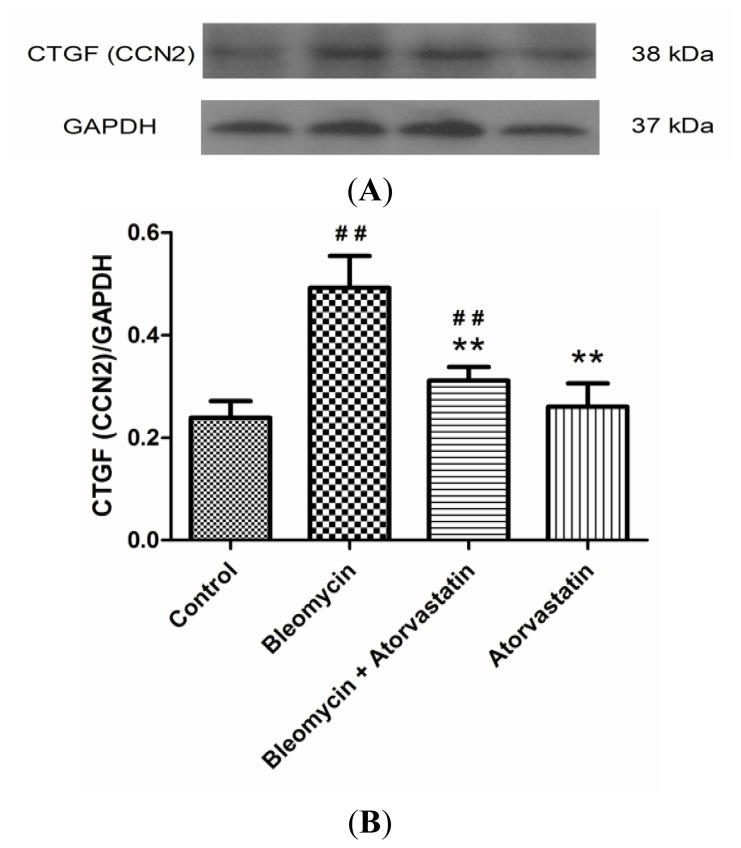
Western blot analysis of CTGF (CCN2) levels in the lungs of bleomycin-induced pulmonary fibrosis rats. The increased levels of CTGF (CCN2) protein expression were obviously suppressed by administration of atorvastatin. (**A**) Representative blots are shown and protein size is expressed in kDa; and (**B**) Densitometric quantification data are expressed as the intensity ratio of target proteins to GAPDH (mean ± SD, *n* = 5). ^##^*p* < 0.01 *vs.* the control group; ** *p* < 0.01 *vs.* the bleomycin group.

CTGF (CCN2) is regarded as one of the key fibrogenic cytokines in the development of pulmonary fibrosis. To analyze the anti-fibrotic efficacy of atorvastatin and to confirm the critical role of CTGF (CCN2), a Western blot analysis for CTGF (CCN2) were performed. The Western blot analysis of the lung tissue of the bleomycin-treated animals showed an increased expression profile of CTGF (CCN2) compared to the control animals. A significant down-regulation of CTGF (CCN2) was observed after the atorvastatin treatments compared to the bleomycin-treated animals (Figure 6A). The quantitative analysis of the Western blots for CTGF (CCN2) is shown in Figure 6B. No significant variations were observed between the control and the animals treated with atorvastatin alone.

### 2.6. Down-Regulation of Phosphorylation of ERK by Atorvastatin Treatment

**Figure 7.**
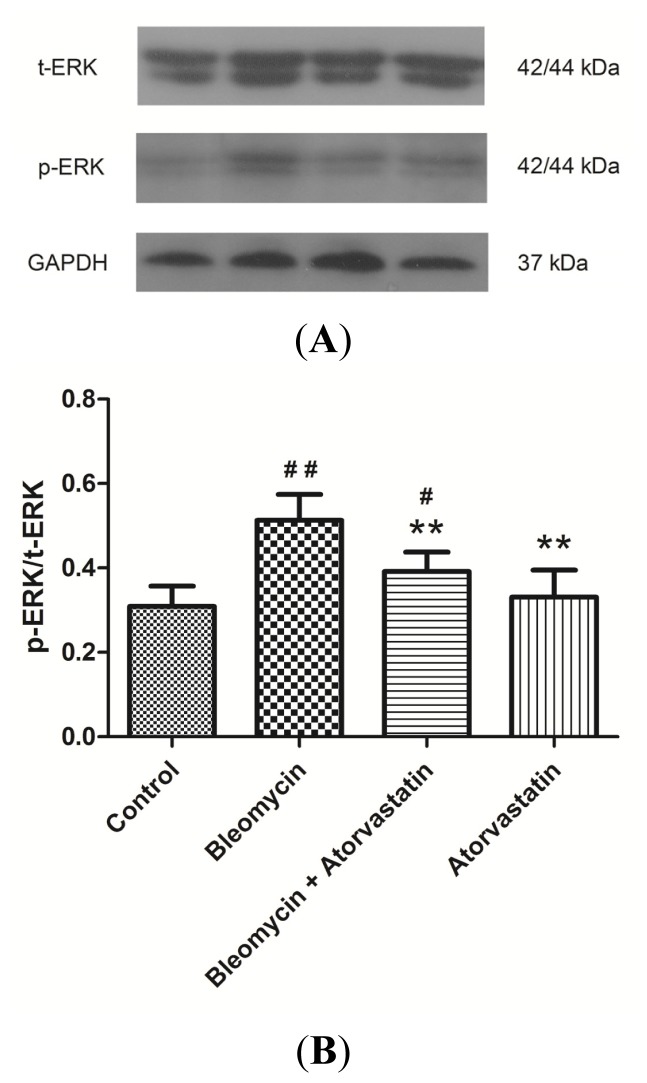
Effects of atorvastatin on the phosphatidylinositol ERK signaling in the lungs of bleomycin-induced pulmonary fibrosis rats. The increased levels of phosphor-ERK were significantly inhibited by administration of atorvastatin. (**A**) Representative blots of phosphor-ERK and total ERK are shown and protein size is expressed in kDa; and (**B**) Densitometric quantification data are expressed as the intensity ratio of target proteins to GAPDH (mean ± SD, *n* = 5). ^#^*p* < 0.05 and ^##^*p* < 0.01 *vs.* the control group, respectively; ** *p* < 0.01 *vs.* the bleomycin group.

We further investigated the possible involvement of the major ERK pathways that have been implicated in fibrosis. As shown in Figure 7, the phosphorylation of ERK was increased in the lung tissues in the bleomycin group compared to the control group, as determined by Western blot analysis, using antibodies against phospho-ERK. Although the total ERK was equal in all samples (Figure 7A), the treatment with atorvastatin and bleomycin showed a remarked reduction in phospho-ERK. However, no significant variations in the phosphorylation of ERK were observed between the control animals and the animals treated with atorvastatin alone (Figure 7B). Together these findings indicated that atorvastatin protects rats against bleomycin-induced pulmonary fibrosis, at least in part, by the inhibiting the CTGF (CCN2)/ERK signaling pathway.

## 3. Discussion

Pulmonary fibrosis is a progressive and fatal lung disease with histopathological characteristics including excessive extracellular matrix (ECM) deposition, patchy chronic interstitial inflammation, fibroblast proliferation, and collapse of alveoli, leading to progressive fibrosis and loss of lung functions [28]. Despite extensive research efforts in experimental and clinical studies, pulmonary fibrosis responds poorly to the available therapy [6]. The development of efficient therapeutic interventions to ameliorate the pathogenic events in pulmonary fibrosis seems to gain significance. The present study shows that atorvastatin attenuates the histological changes in pulmonary fibrosis induced by bleomycin in rats. The data further show that atorvastatin reduces the levels of hydroxyproline, MDA and NO induced by bleomycin. The molecular evidence indicates that iNOS and the CTGF (CCN2)/ERK pathway are inhibited by atorvastatin treatment.

Bleomycin, a glycopeptide antibiotic, has been used clinically for a variety of cancers [29]. As a side effect of its therapeutic use, bleomycin causes destruction of the lung architecture, leading to pulmonary fibrosis that is characterized by an increase in hydroxyproline levels and collagen deposition in the lungs [30]. Bleomycin-induced lung fibrosis is a widely used animal model of human idiopathic pulmonary fibrosis [31,32]. The route of intratracheal instillation generally causes an inflammatory response and increased epithelial apoptosis for the first seven days, closely resembling acute lung injury. These effects are followed by three days of transition, in which the inflammation resolves and fibrosis is detected. The fibrotic stage persists until three to four weeks after the bleomycin instillation, and is characterized by the excessive deposition of extracellular matrix, causing areas of fibrosis [33,34]. The present study showed a substantially increased intensity of collagen in the bleomycin-treated animals, which reflected the detrimental alterations associated with fibrosis. The amplified hydroxyproline levels correlates with the accumulated collagen in the alveolar space. Our data also indicated that the atorvastatin-exposed rats exhibited significantly lower lung weight and lung indices (lung/body weight). The ameliorating effects of atorvastatin on histological alterations might be due to their radical scavenging activities, which prevent the accumulation of hydroxyproline in bleomycin-induced lung tissues.

Growing evidence indicates the essential role of oxidative stress in the pathophysiology of many diseases, including lung fibrosis [8,35]. Reactive oxygen species (ROS) and reactive nitrogen species (RNS) were reported to increase in bleomycin-induced pulmonary fibrosis in animals [12]. Recently, extensive focus has been shifted to RNS. Nitric oxide (NO), an endogenous short-lived free radical, is synthesized by three isoforms of NO synthase (NOS): neuronal (nNOS), endothelial (eNOS), and inducible (iNOS) isoforms [36]. Previous studies have demonstrated that an overproduction of NO, resulting from the expression of iNOS, appears in pulmonary fibrosis in both animal models and humans [15,16]. In this study, we found that bleomycin induced a marked increase in lipid peroxidation as indicated by the MDA level, and this level was significantly decreased upon atorvastatin supplementations. In addition, atorvastatin treatments significantly attenuated the bleomycin-mediated oxidative stress, as indicate by the decreased levels of NO. The Western blot analysis showed that atorvastatin treatment strikingly attenuated the increase of iNOS protein in the lung tissue of the bleomycin-treated animals. These results indicated that atorvastatin could attenuate oxidative stress via suppressing iNOS expression in bleomycin-induced pulmonary fibrosis.

CTGF (CCN2), a cysteine-rich 38 kDa secreted protein, has been proven to play an essential role in the fibrogenetic processes of the lung, kidney, and liver [37–39]. As a downstream mediator of transforming growth factor beta (TGF-β), CTGF is considered a prognostic marker in evolving pulmonary fibrosis and may result in collagen overproduction and deposition in the lung [40]. Elevated expression of CTGF and TGF-β1 has also been found in the lung tissues of patients and animals with pulmonary fibrosis [41]. Our findings suggest that atorvastatin may down-regulate CTGF (CCN2) protein expression, thus, modulating the collagen synthesis in bleomycin-induced pulmonary fibrosis. The above two factors, iNOS and CTGF (CCN2), play critical roles during the pulmonary fibrosis induced by bleomycin, and, therefore, have been regarded as pathological markers in researches and as potential therapeutic targets in treatments.

Although there are some reports on the therapeutic potential of statins for pulmonary fibrosis, the underlying mechanisms for the statin-mediated regulation of oxidative stress in pulmonary fibrosis have not been elucidated. The mitogen-activated protein kinase (MAPK)/extracellular signal-regulated kinase (ERK) signaling cascade is a major pathway controlling cellular processes associated with fibrogenesis, including growth, proliferation, and survival. It has been shown that statins inhibited the MAPK signaling pathway in vascular smooth muscle cells, which regulates the AngII/Smad pathway and related profibrotic factors and matrix proteins [42]. Recent evidence has shown that ERK activation is increased in the bleomycin model of lung fibrosis [43]. The Western blot of human lung biopsy samples also demonstrates increased ERK1/2 signaling in idiopathic pulmonary fibrosis samples [44]. Our results show that the increased level of ERK phosphorylation after bleomycin stimulation is inhibited by atorvastatin administration. Moreover, the decrease in ERK phosphorylation is correlated with the reduction of CTGF (CCN2) expression. These findings suggest that atorvastatin may inhibit ERK activation, thus, reducing the expression of the fibrogenic cytokine CTGF (CCN2) in bleomycin-induced oxidative stress.

The optimal therapy for pulmonary fibrosis remains controversial. No agent has been rigorously shown to improve the survival or quality of life for patients with pulmonary fibrosis [45]. Lung transplantation is presently the only effective therapy for pulmonary fibrosis [46]. However, lung transplantation has many limitations attributable to organ shortages and complications associated with long-term immunosuppression [47]. Therefore, the development of effective therapies to reduce or reverse pulmonary fibrosis is important clinically for reducing the morbidity and mortality associated with pulmonary fibrosis and the need for lung transplantation. The clinical ineffectiveness of corticosteroids and immunosuppressives/cytotoxics, clarification of the unique histopathologic features of pulmonary fibrosis, and a greater understanding of the injury/repair microenvironment of the lung have led to the proposal that pulmonary fibrosis results from sequential lung injury and subsequent aberrant wound healing, thus, generating interest in agents that specifically alter fibroproliferative mechanisms. The attention in pulmonary fibrosis pathogenesis research has shifted from chronic inflammation to aberrant wound/repair mechanisms, with increased emphasis on the interplay of fibroblasts and alveolar epithelial cells [48]. Consequently, the search for therapies has turned from traditional anti-inflammatory agents to those that may modify the cellular and cytokine constituents of fibroproliferative processes [49]. Statins are pharmacologic agents that are widely used in the treatment of hypercholesteremia. In addition to their immunomodulatory and antiinflammatory properties, statins can also be effective antifibrotic agents because they exhibit antifibrotic efficacy by modulating some fibrogenic cytokines in the development of pulmonary fibrosis. However, other data has showed that statins are not beneficial for survival in patients with idiopathic pulmonary fibrosis and attenuate decline in lung function in the elderly [50,51]. This result suggests statins could be used to treat impaired lung function; however, this possibility requires further investigation.

## 4. Experimental Section

### 4.1. Animal and Maintenance

Male Sprague-Dawley rats (six weeks old, 180–220 g) were obtained from the Experimental Animal Center of Xi’an Jiaotong University, and were housed in separate cages under standard temperature (25 ± 2 °C) and 12 h light/dark photoperiod. The rats were acclimatized for three days before the start of the experiment and provided food and water *ad libitum*. The experiments were designed and conducted according to the guidelines for the Care and Use of Laboratory Animals issued by the Chinese Council on Animal Research and the Guidelines of Animal Care [52]. All animal experimentation protocols were approved by the Ethical Committee of Xi’an Jiaotong University.

### 4.2. Experimental Protocol

An animal model of bleomycin induced pulmonary fibrosis as reported earlier, was used in this study [53]. Briefly, after recording the body weights, the rats were anesthetized via sn intraperitoneal injection of sodium pentobarbital (40 mg/kg). The skin and subcutaneous tissue overlying the proximal portion of the trachea were exposed by blunt dissection. A single intratracheal instillation of 1 mg/kg of bleomycin (Sigma-Aldrich, St. Louis, MO, USA) in sterile 0.9% NaCl was administered to the rats to develop the model for pulmonary fibrosis. The rats in the control group and the atorvastatin (Pfizer Pharmaceutical Co. Ltd, New York, NY, USA) group were given a single intratracheal dose of sterile saline alone.

Thirty-two rats were randomly divided into four experimental groups with eight animals each. The control group animals received an intratracheal injection of normal saline alone. The bleomycin group animals were subjected to a single intratracheal instillation of bleomycin as previously mentioned. The bleomycin plus atorvastatin group of animals were considered as a preventive model, which received the same amount of bleomycin as the bleomycin group animals and were then treated with atorvastatin (10 mg/kg intragastrically) for 28 days. The atorvastatin alone group received an intratracheal injection of normal saline and was then treated with atorvastatin (10 mg/kg intragastrically) for 28 days.

Twenty-eight days after the bleomycin treatment, the animals were sacrificed, and their body weights and lung weights were recorded. The lung tissues were divided into pieces, one part was immersed in 10% formalin solution for histopathological examination, one part was immersed in liquid nitrogen for Western blot analysis, and the remaining part was immersed in 0.9% saline to obtain the tissue homogenate. The lung tissue homogenates were prepared in appropriate homogenizing buffer and stored at −80 °C as aliquots for further assay.

### 4.3. Histological Examination and Masson’s Trichrome Staining

After sacrificing the animals, one part of the lungs was carefully excised and fixed for one week in 10% (*w*/*v*) PBS-buffered formaldehyde solution at room temperature, dehydrated using graded ethanol and embedded in paraffin. The paraffin-embedded tissues were cut to 5 μm thicknesses with a microtome (RM-2135, Leica Microsystems, Bensheim, Germany). To evaluate the histopathological changes, the sections were subjected to haematoxylin and eosin staining. To identify the density of the accumulated collagen fibers Masson’s trichrome staining was performed.

### 4.4. Estimation of Hydroxyproline (HYP)

The collagen content was determined by measuring the HYP levels, as described previously [54]. Briefly, 10 mg of lung tissues were minced in 1 mL 6 mol/L HCl, hydrolyzed and incubated overnight at 120 °C. Citric/acetate buffer was added and the pH was adjusted between 6.0 and 6.5 with 0.2 mol/L of NaOH. 1 mL of chloramine T solution (0.05 mol/L) was added and incubated for 20 min at room temperature. One milliliter of aldehyde-perchloric acid was added, incubated at 60 °C for 15 min, and the absorbance of each sample was recorded at 550 nm. The results were calculated as μg/mg wet lung weight using the standard curve of hydroxyproline.

### 4.5. Measurements of Malondialdehyde (MDA) and Nitric Oxide (NO) Level

The lung tissue homogenates of the control and experimental groups of animals were prepared with 0.1 M Tris–HCl buffer (pH 7.4) at 4 °C. The resulting tissue homogenates were used for biochemical measurements. The contents of MDA and NO were determined using colorimetric assay with commercially available kits (Jiancheng Bioengineering Institute, Nanjing, China) according to the manufacturer’s instructions. Briefly, the production of NO was determined using nitrate reductase to specifically reduce nitrate to nitrite; the latter was quantified by a colorimetric assay. The absorbance was determined at 550 nm on a Stat Fax 2100 spectrophotometer (Awareness Technology Inc., Palm City, FL, USA). The MDA content in the serum was measured using the thiobarbituric acid method, based on the formation of a red complex when MDA reacts with thiobarbituric acid. The absorbance was measured spectrophotometrically at 532 nm.

### 4.6. Western Blot Analysis

The lung tissues were homogenised in RIPA lysis buffer (Beyotime Biotech, Haimen, China) containing 1 mM phenylmethylsulphonyl fluoride. The lysates were then centrifuged at 12,000 rpm for 15 min at 4 °C, and the supernatants, which contained the tissue protein extracts, were collected and stored at −80 °C. The total protein concentrations were determined using the Bicinchoninic acid protein assay kit (Beyotime Biotech, Haimen, China). The samples (30 μg) were mixed with sample buffer, denaturated by heating at 95 °C for 5 min, resolved via SDS-PAGE and transferred to nitrocellulose membranes. The membranes were blocked with 5% non-fat dried milk in Tris-buffered saline with Tween (TTBS) for 1 h and probed with primary antibodies against iNOS (1:1000 dilution, Santa Cruz, CA, USA), CTGF (CTGF; 1:1000 dilution, Santa Cruz, CA, USA), ERK (1:1000 dilution; Beyotime Biotech, Haimen, China), and phospho-ERK (1:1000 dilution; Beyotime Biotech, Haimen, China) overnight at 4 °C. Next, they were washed with TTBS and incubated with horseradish peroxidase-conjugated secondary antibodies (1:5000; Cowin Biotech, Beijing, China) for 1 h. Finally, the blots were developed with ECL-Plus reagent (Millipore, Bedford, MA, USA) and the graphs were analyzed by the Gel-Pro Analyzer (Media Cybernetics, Bethesda, MD, USA).

### 4.7. Statistical Analysis

The data are presented as the means ± SD (standard deviation), and n indicates the number of animals studied. The statistical analysis was performed using Student’s t test or a one-way analysis of variance, followed by the Tukey *post hoc* test for multiple comparisons. All the statistical analyses used SPSS software (SPSS, Chicago, IL, USA). *p* < 0.05 was considered statistically significant.

## 5. Conclusions

In the present study, we demonstrated the anti-fibrotic efficacy of atorvastatin against bleomycin-induced pulmonary fibrosis. Briefly, atorvastatin attenuated bleomycin-induced oxidative stress, histological alterations and collagen depositions via the inhibition of the pro-fibrotic cytokine iNOS, CTGF (CCN2) expression and the ERK pathway. Our results suggest that the pleiotropic actions of atorvastatin might be utilised in combination therapy for pulmonary fibrosis in clinics.

## Figures and Tables

**Table 1.** Effect of atorvastatin on body weight and lung indices of bleomycin-induced pulmonary fibrosis rats.

Group	Initial Body weight (g)	Final Body Weight (g)	Lung Weight (mg)	Lung Indices (mg/kg)
Control	208.9 ± 7.8	304.9 ± 15.1	1,393.8 ± 129.9	4,565.5 ± 257.6
Bleomycin	208.0 ± 9.7	283.8 ± 13.6 #	2,190.0 ± 336.2 ##	7,714.1 ± 1,104.0 ##
Bleomycin + Atorvastatin	208.8 ± 9.5	290.5 ± 17.3	1,930.0 ± 200.1 ##,*	6,633.7 ± 402.2 ##,*
Atorvastatin	208.3 ± 10.0	292.5 ± 12.1	1,366.3 ± 116.6 **	4,664.5 ± 231.1 **

Values are given as mean ± SD for groups of eight rats each.

# *p* < 0.05 and

## *p* < 0.01 *vs.* the control group, respectively;

* *p* < 0.05 and

** *p* < 0.01 *vs.* the bleomycin group, respectively.
